# Neuromuscular organization of the benthic ctenophore, *Vallicula multiformis*

**DOI:** 10.1186/s40851-024-00225-0

**Published:** 2024-01-30

**Authors:** Kurato Mohri, Hiroshi Watanabe

**Affiliations:** Evolutionary Neurobiology Unit, Okinawa Institute of Science and Technology Graduate University, 1919-1 Tancha, Onna-son, Kunigami-gun, Okinawa, 904-0495 Japan

**Keywords:** Ctenophora, *Vallicula multiformis*, Neurons, Muscles, Neuropeptides

## Abstract

Ctenophora is the earliest metazoan taxon with neurons and muscles. Recent studies have described genetic, physiological, and cellular characteristics of the neural and muscular systems of this phylogenically important lineage. However, despite the ecological diversity of ctenophore niches, including both pelagic and benthic forms, studies have focused predominantly on pelagic species. In the present study, we describe the neural and muscular architectures of the benthic ctenophore, *Vallicula multiformis* (Order Platyctenida), employing immunohistochemical analysis using antibodies against amidated neuropeptides with the C-terminal sequences VWYa, NPWa, FGLa, or WTGa to compare it to pelagic species. In *V. multiformis*, which lacks the characteristic comb rows seen in pelagic ctenophores, neural structures that develop beneath the comb were not detected, whereas the subepithelial and tentacle neural networks showed considerable similarity to those of pelagic species. Despite significant differences in morphology and lifestyle, muscle organization in *V. multiformis* closely resembles that of pelagic species. Detailed analysis of neurons that express these peptides unveiled a neural architecture composed of various neural subtypes. This included widely distributed subepithelial neural networks (SNNs) and neurosecretory cells located primarily in the peripheral region. The consistent distribution patterns of the VWYa-positive SNN and tentacle nerves between *V. multiformis* and the pelagic species, *Bolinopsis mikado*, suggest evolutionarily conserved function of these neurons in the Ctenophora. In contrast, NPWa-positive neurons, which extend neurites connecting the apical organ and comb rows in *B. mikado*, showed a neurite-less neurosecretory cell morphology in this flattened, sessile species. Evaluation of characteristics and variations in neural and muscular architectures shared by benthic and pelagic ctenophore species may yield valuable insights for unraveling the biology of this rapidly evolving yet enigmatic metazoan lineage. These findings also provide important insight into neural control modalities in early metazoan evolution.

**Supplementary Information:**

The online version contains supplementary material available at 10.1186/s40851-024-00225-0.

## Background

The phylum Ctenophora (comb jellies) comprises about 200 species [1–3]. Ctenophores have gelatinous bodies and are characterized by eight ciliated comb rows. Most species in this phylum are pelagic, but species belonging to the order Platyctenida are benthic as adults [4–7]. A growing body of evidence suggests that Ctenophora is the phylogenetically oldest branch of the Metazoa; therefore, ctenophores are a key animal lineage for reconstructing genetic, physiological, and anatomical features of ancestral animals [8–11]. Genomic analysis of the ctenophores, *Pleurobrachia bachei* and *Mnemiopsis leidyi*, showed that they lack neuron- and muscle-associated genes generally conserved in other animals having these tissues [8, 9]. For example, they do not have neuronal genes encoding certain important transcription factors that control neuronal cell fate and patterning in bilaterians, and they lack receptors and synthetic enzymes for chemical neurotransmitters, such as acetylcholine and monoamines [8, 9]. In addition, key transcription factors required for mesodermal specification and muscle differentiation such as *twist, NK2.1, par3/7*, and *myoD* appear to be absent [8, 9]. The paucity of distinct neuron-related markers in ctenophores and the presence of unique neural traits such as subepithelial syncytial neural architecture [12] have been interpreted as evidence that the ctenophore nervous system was acquired independently from those of cnidarian and bilaterian lineages.

**Fig. 1:**
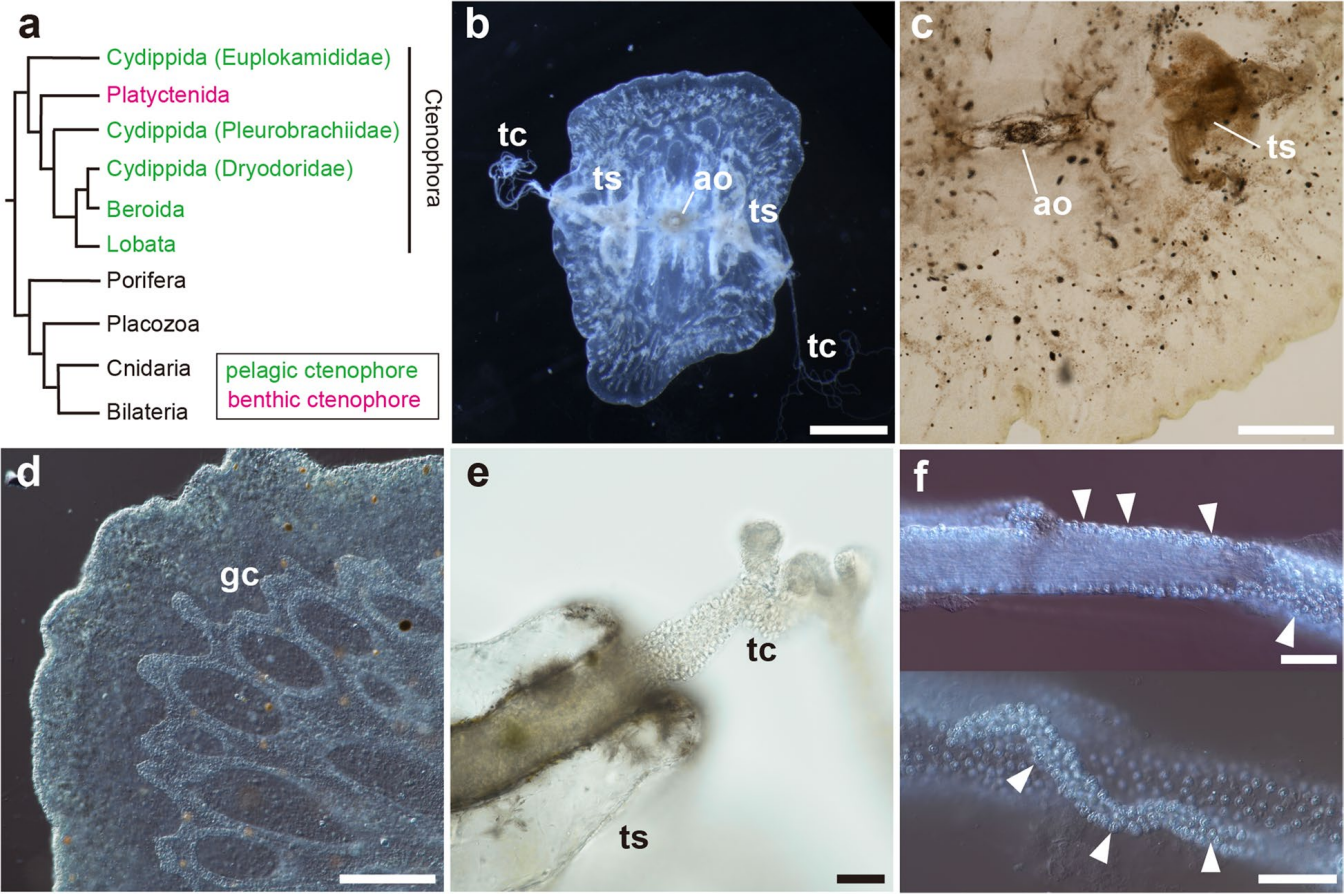
A benthic ctenophore, *Vallicula multiformis*. **a** A phylogenic tree indicating relationships of metazoan phyla and those of the orders of Ctenophora (redrawn and modified from Whelan et al. 2017 [10]). The phylum Ctenophora is the earliest branch of the Metazoa. Most ctenophore species are pelagic, whereas those belonging to the Platyctenida are benthic as adults. **b** An aboral view of an adult *V. multiformis*. It attaches to substrates with its flat body and captures prey using a pair of tentacles (tc). **c** Magnified brightfield image of the aboral surface. The position of the aboral organ (ao) (also called apical organ) and tentacle sheathes (ts) are indicated. **d** The DIC image of the peripheral end of the body. The internal branching gastrovascular canal (gc) is observed. **e** The magnified brightfield image of the aperture of the tentacle sheath and elongating tentacles. **f** The DIC images of the main trunk (top) and tentilla (bottom) of the tentacle. The surface of the tentacles is covered with colloblasts (arrowheads), the ctenophore-specific adhesive cells used for capturing prey. Scale bar, 1 mm (**b**), 500 μm (**c**), 200 μm (**d**) and 50 μm (**e**, **f**)

Recently, there have been significant advances in understanding structural and physiological features of ctenophore neurons. Employing mass spectrometry-based peptidomics, a substantial number of short amidated peptides has been identified from *Bolinopsis mikado* [13]. Some of these peptides correspond to products of genes previously predicted to encode peptides [14]. Experiments in which *B. mikado* cydippid larvae were treated with synthetic mature neuropeptides revealed the functional involvement of neuropeptides in muscle contraction [13]. These findings clearly demonstrate the prevalence of peptidergic neurons across the Metazoa, including the Ctenophora, and provide valuable insights into the most ancient nervous systems. Anatomical investigations of neuronal and muscular structures in Ctenophora relied predominantly on classical methodologies like methylene-blue staining and electron microscopic analysis [1, 15–20]. More recently, immunohistochemical analysis utilizing anti-α-tubulin antibodies and phalloidin has shed light on cytological intricacies of neuron and muscle structures in both adult and embryonic stages of various pelagic species. This approach has included taxa such as *P. bachei*, *P. pileus*, *Hormiphora hormiphora* (Cydippida Pleurobrachiidae), *Dryodora glandiformis* (Cydippida Dryodoridae), *Euplokamis dunlapae* (Cydippida Euplokamididae), *Beroe abyssicola* and *B. ovata* (Order Beroida), *B. infundibulum* and *M. leidyi* (Order Lobata) (Fig. 1a) [9, 21–26]. However, anatomical traits of benthic species are still undescribed. The Order Platyctenida is the only benthic group in the phylum Ctenophora, comprising five distinct families (Coeloplanidae, Ctenoplanidae, Tjalfiellidae, Lyroctenidae, and Savangiidae) and about 50 species [4–7] (Fig. 1a). They exhibit flattened body shapes, devoid of ciliary comb plates, and attach to substrates, including rocks, plants, or sessile marine organisms [4, 6, 27, 28].

In the present study, we employed immunohistochemical staining techniques to visualize microtubules and actin fibers in the ctenophore species, *Vallicula multiformis* (Order Platyctenida) (Fig. 1b). Our objective was to describe architectural intricacies of neurons and muscles in benthic ctenophores and to compare them with anatomical features observed in pelagic ctenophores. Furthermore, through a comprehensive analysis of the morphology and arrangement of peptidergic neurons in *V. multiformis*, we uncovered both functional commonalities and distinctive features associated with these peptides in the Ctenophora.

## Results

### Subepithelial neural networks and aboral organ of *V. multiformis*

**Fig. 2:**
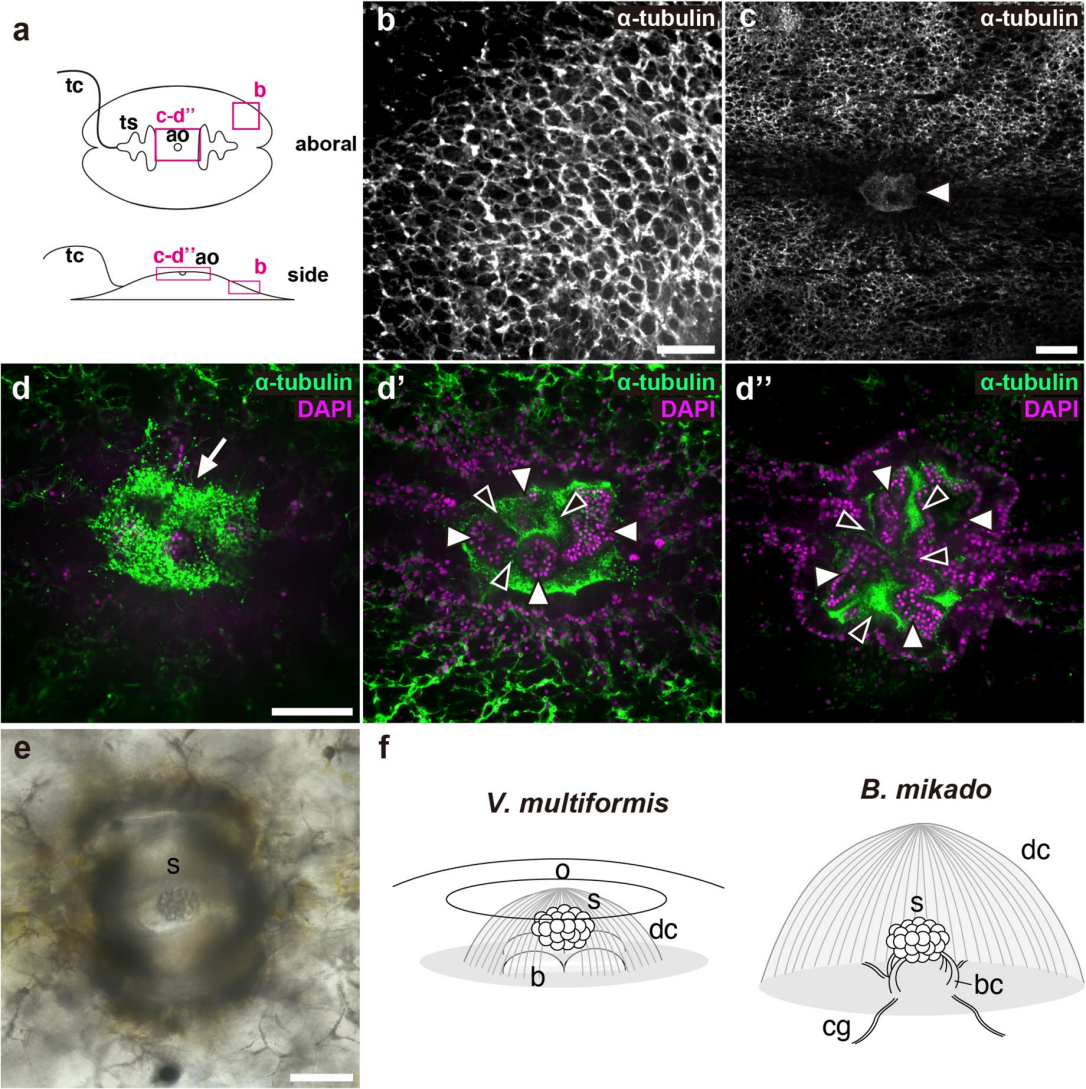
Aboral nervous system of *V. multiformis.* Immunofluorescent staining was performed with an anti-α-tubulin antibody to observe nervous systems of *V. multiformis*. **a** Positions of the following images in the animal are indicated. **b**, **c** Subepithelial neural-net-like structures of the peripheral (**b**) and central part (**c**) of the aboral surface. The arrowhead indicates the aboral organ. **d**-**d’’** A series of images from external (**d**) to internal (**d’’**) focal planes of the aboral organ, stained with anti-α-tubulin antibody (green) and DAPI (magenta). The arrow indicates the cilia covering the internal organ, presumed to be dome cilia. The white and black arrowheads indicate the epithelial bulges and cilia surrounding these bulges, respectively. **e** A light microscopy image of the statolith of the living *V. multiformis*. **f** The schematic drawing of the aboral organ of *V. multiformis* reconstructed from immunofluorescent images and the pelagic ctenophore *B. mikado *(modified from Jokura and Inaba 2020 [29]) for comparison. The position of the statolith in *V. multiformis* is depicted based on that of *B. mikado* and other ctenophores. b, bulge; bc, balancer cells; cg, ciliated groove; dc, dome cilia; o; opening; s, statolith. Scale bar, 50 μm

To gain insight into the neural architecture of *V. multiformis* we first sought to describe the microtubular network. A recent neuroanatomical study on ctenophores employed anti-tyrosinated-α-tubulin antibodies to visualize neuronal processes [9, 21–26]. However, our attempts yielded only partial and faint signals in *V. multiformis* (data not shown). Consequently, we employed anti-α-tubulin antibodies, which stained the network in the subepithelial layer on the aboral side (Fig. 2). This staining pattern resembled subepithelial neural networks (SNN) observed in other ctenophore species [9, 21, 23–25]. However, the mesh-like staining pattern was thicker than those observed in other ctenophore species.

The organ, inside the opening at the center of the aboral surface was identified as the aboral organ of *V. multiformis*, also known as the apical organ in pelagic species [6] (Fig. 2c). α-tubulin staining visualized numerous cilia covering the four epithelial bulges (Fig. 2d-d”, white arrowheads), which are presumed to be dome cilia (Fig. 2d, arrow), akin to those observed enveloping the internal statolith in other ctenophore species [29, 30]. Inside the organ, cilia surrounding epithelial bulges were also found (Fig. 2d’-d”, black arrowheads). We did not detect specific cells such as the statolith and balancer cells required for sensing gravity in pelagic ctenophores. The presence of the statolith in the aboral organ was previously reported in *V. multiformis* [6]. It was lost during fixation and immunohistological processing, as has been reported in other ctenophores [23], but we confirmed that the statolith is present in living animals (Fig. 2e). In the pelagic ctenophores, the neural structure called the ciliated groove (also known as the ciliated furrow) runs from the apical organ to each comb-row (Fig. 2f right); these structures were not observed in *V. multiformis*. We also did not observe mesogleal neurons that connect the external surface and internal organs and that run across the mesogleal space, which are conserved in pelagic species [9, 21, 23–26].

### Body muscles of *V. multiformis*

**Fig. 3:**
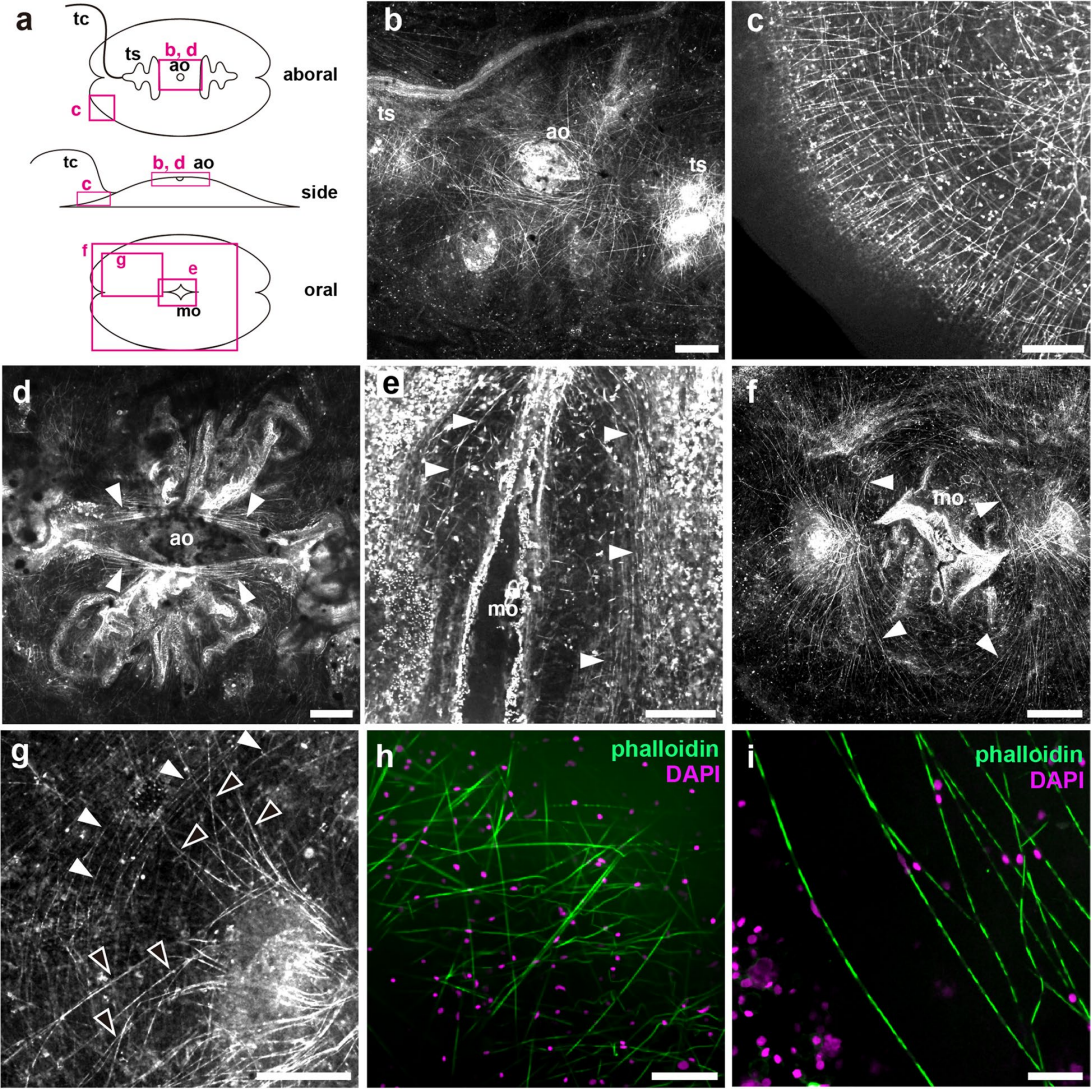
Muscular organization of *V. multiformis*. **a** A schematic representation indicating positions of the following images. **b**–**g** Muscular fibers of the body of *V. multiformis* were visualized by phalloidin staining. **b** Randomly oriented muscular fibers that were observed on aboral subepithelial layers. **c** High-magnification view of the crossing longitudinal fibers and circular muscle fibers on peripheral aboral surfaces. **d** Parallel bundles of muscle fibers run along the aboral organ (ao) (arrowheads). **e** Thin muscle fibers encircling the mouth openings (mo) on the oral subepithelial layers (arrowheads). **f** Radially elongated thicker fibers on the oral side (arrowheads indicating representative fibers). **g** Crossing of radial thick fibers (black arrowheads) and circular fibers (white arrowheads) observed on the oral side of the body. **h**, **i** High-magnification images of muscle fibers stained with phalloidin (green) and DAPI (magenta). Randomly oriented fibers on aboral surfaces (**h**) and striated thick fibers of radial muscle fibers on the oral side (**i**). Scale bar, 100 μm (**b**–**g**) or 20 μm (**h** and **i**)

The fibrous structures that exhibit robust staining with phalloidin, a marker for F-actin, are thought to be muscles [22–26, 31]. On the aboral side of the body, thin fibers form a reticular structure beneath the epithelium. A profusion of these fibers is concentrated around the aboral organ and sheaths of tentacles (Fig. 3b). These fibers appear to be arranged as radial muscles, emanating from the center toward the exterior, with parietal circular muscles running orthogonally to the radial muscles (Fig. 3c). In the central region, two parallel bundles of thicker fibers connect tentacle sheaths, with the aboral organ nestled among these bundles (Fig. 3d, arrowheads). On the oral side, beneath the oral epithelial layer, circular muscles composed of concentric thin filaments were observed. In the center, they surround the mouth opening (Fig. 3e, arrowheads). Further inside, long, radially spread thick fibers were observed (Fig. 3f). These fibers extend from areas of both tentacle sheaths to the body surface. Exteriorly on the oral side, circular muscles and thick radial muscles intersect (Fig. 3g). A schematic overview of these muscle fibers observed in *V. multiformis* is shown in Fig. 11a. The thickness of muscle fibers varies, with the thickest being 1.2 μm (*n* = 10, number of animals examined). A striated pattern was observed in some of these fibers, especially the thick ones (Fig. 3h, i). The pitch of the striated patterns was wide and uneven, suggesting that these fibers represent a structure distinct from the typical striated muscles of bilaterian animals [32]. Nuclei of muscle cells were located mainly, but not exclusively, in phalloidin-negative regions (Fig. 3i).

### Tentacles

**Fig. 4:**
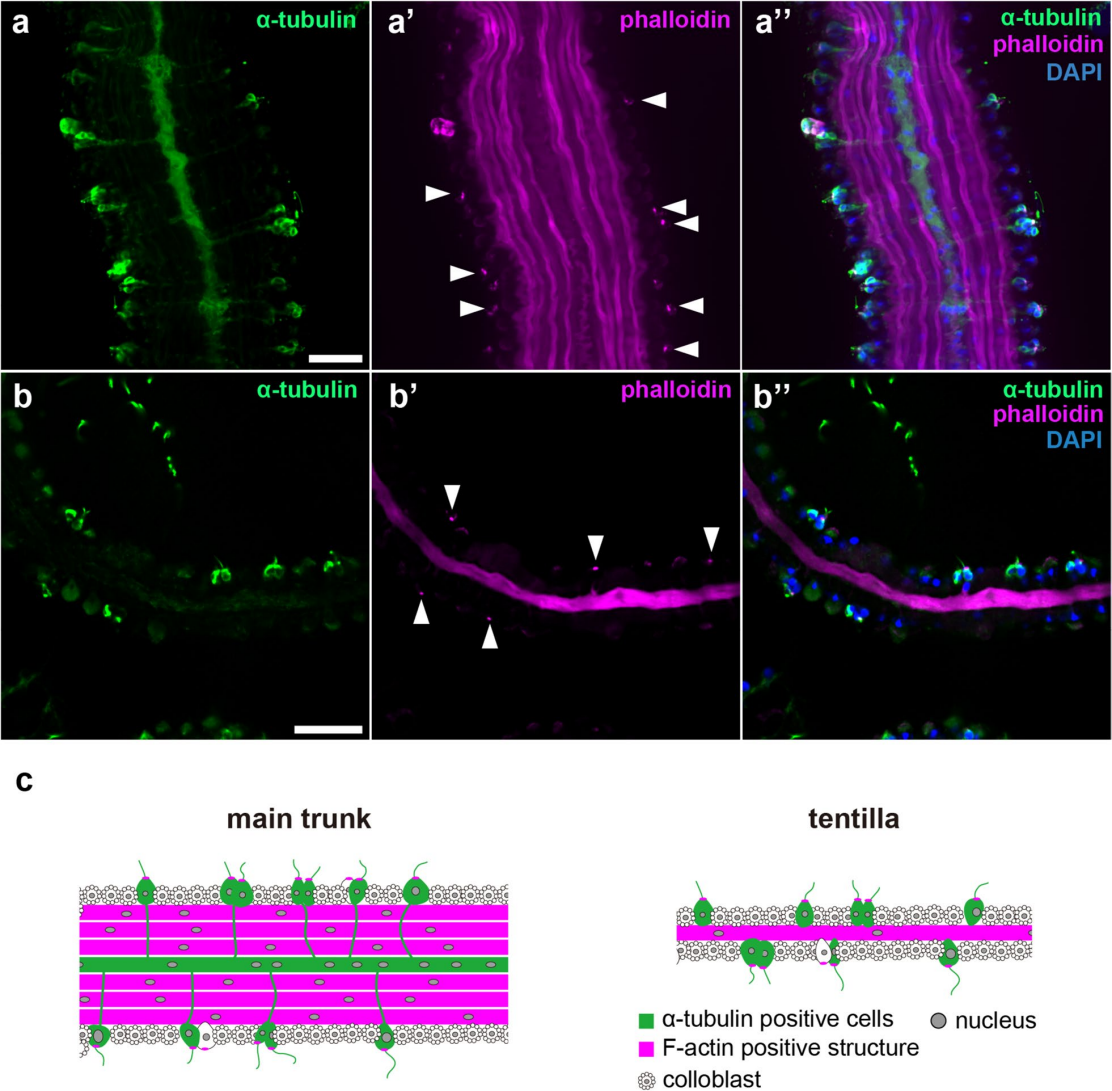
Neural and muscular organizations of *V. multiformis* tentacles. The main trunk (**a**-**a”**) and tentilla (**b**-**b”**) were stained with anti-α-tubulin antibody (green), phalloidin (magenta), and DAPI (blue). **a**-**a”** The α-tubulin positive central fiber and putative sensory neurons that extend neurites toward central fibers and bundles of phalloidin-positive thick muscular fibers were observed in the main trunk. **b**-**b”** Only the cell bodies of putative sensory cells were positive for α-tubulin; no neurite-like extensions were observed in tentilla. Muscle of the tentilla was a thick, single fiber. Arrowheads indicate phalloidin-positive projections of putative sensory cells on the surfaces. **c** The schematic drawings of tentacles reconstructed from the immunostaining results. Scale bar, 20 μm

*Vallicula multiformis* uses a pair of long tentacles to capture prey, each comprising a thick main trunk bearing an array of slender tentilla [6, 33]. Anti-α-tubulin staining of the main trunk demonstrated enrichment of the tubulin signal through the center, as well as neuronal cell bodies embedded in the outer epithelium and their neurites extending inward into the tentacles (Fig. 4a, a’’). On the other hand, in the tentilla, although sensory cell bodies were observed in the epithelium, nerve fibers were absent (Fig. 4b-b’’). α-tubulin-positive cells on the surface have a single α-tubulin-positive cilium with accumulation of F-actin at the base (Fig. 4), suggesting sensory cell function.

There were muscle fibers at the center of both the main trunk and tentilla. Aligned muscle fibers, thicker than those of the body muscle, were observed in the center of the main trunk and one thick fiber of each tentillum (Fig. 4). These neural and muscular structures in the main trunk resemble those described in tentacles of *P. bachei* [23].

### Mouth and oral epithelium

**Fig. 5:**
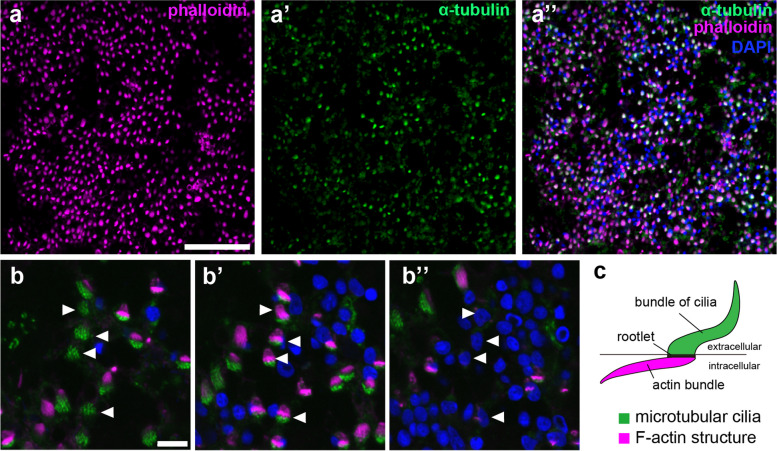
Ciliated structures in oral epithelium of *V. multiformis*. **a**-**a”** The oral surface of *V. multiformis* was stained with phalloidin (**a**) and anti-α-tubulin antibody (**a’**) and merged (**a”**). **b**-**b”** A magnified view of oral surface cells. A series of images from external (**b’**) to internal (**b”**) focal planes. Arrowheads indicate positions of representative cells for comparison in different focal planes. **c** The schematic drawings of the pharyngeal cilia, a characteristic structure observed on the pharyngeal surfaces of pelagic ctenophores [23–25, 34]. The pharyngeal cilia are composed of the external microtubular cilia and the intracellular F-actin bundle. Colors in all images indicate anti-α-tubulin (green), phalloidin (magenta), and DAPI (blue) fluorescence. Scale bar, 50 μm (**a**) or 5 μm (**b**)

Benthic ctenophores attach to substrates via the oral surface with a central mouth opening [4–6]. Immunostaining with anti-α-tubulin staining revealed tubulin-positive bundles of cilia covering the entire oral epithelium and phalloidin-positive F-actin bundles were closely associated with these tubulin-positive structures (Fig. 5a–a”). Distributions of these α-tubulin and phalloidin signals exhibited remarkable proximity, with α-tubulin-positive cilia extended as the outermost parts and connected with phalloidin-positive structures at their bases (Fig. 5b–b”). These cilia, which connect with actin bundles at their roots, were initially described as pharyngal cells in *Beroe* sp. and named macrocilia [34]. Similar pharyngeal cells were subsequently found in most ctenophore species examined, such as *E. dunlapae*, *P. bachei*, *H. hormiphora*, *Beroe abyssicola* and *Bolinopsis infundibulum* [23–25]. These results suggest that the oral surface of *V. multiformis* is homologous to the outer surface of the pharynx of pelagic ctenophores.

### Gastrovascular canal

**Fig. 6:**
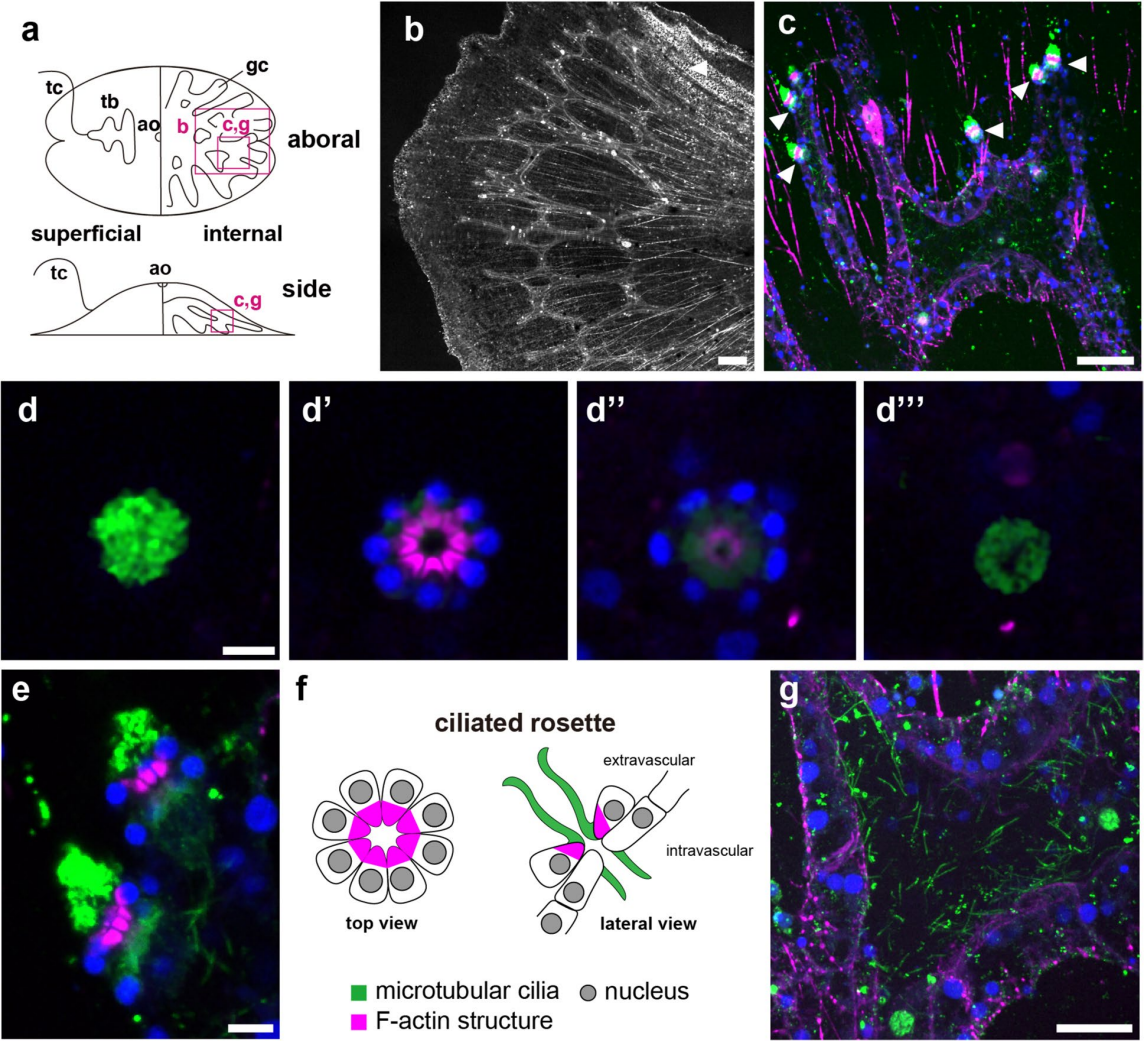
The gastrovascular canal of *V. multiformis*. **a** A schematic representation indicating positions of the following images. **b** The gastrovascular canal of *V. multiformis.* Radiating canals with multiple branches were observed with phalloidin staining. **c** A gastrovascular canal was stained with an anti-α-tubulin antibody, phalloidin and DAPI. Arrowheads indicate the ciliated rosette. **d**-**d’’’** A series of images from external (**d**) to internal (**d’’’**) focal planes of one ciliated rosette. **e** The lateral view image of the two ciliated rosettes. **f** The schematic drawings of the ciliated rosette reconstructed from the immunofluorescent images. **g** A magnified view of the canal. α-tubulin-positive cilia covered the inner surface. Colors in all images except **b** indicate anti-α-tubulin (green), phalloidin (magenta), and DAPI (blue) fluorescence. Scale bar, 20 μm (**b**, **c** and **g**) or 5 μm (**d**, **e**)

In addition to muscular structures, the configuration of the gastrovascular canal was visualized with phalloidin staining (Fig. 6b, c). An intricately convoluted stomach is connected to the mouth openings in the central region. These canals radiate outward with multiple branching patterns throughout the body (Fig. 6b). In pelagic ctenophores, gastrovascular canals extend to the aboral epithelium and are lined with comb rows as meridional canals [2]. In contrast, *V. multiformis* lacks comb rows, and canals are not situated beneath the aboral epithelium. We observed a ciliated rosette on the surface of gastrovascular canals (Fig. 6c, e). A rosette consists of two layers of eight radially arranged cells bearing long, microtubular cilia (Fig. 6d-d’’’). Rosettes are widely distributed on surfaces of meridional canals of pelagic ctenophores such as *P. bachei, Beroe* sp., *B. abyssicola* and *E. dunlapae* and the proposed function of these structures involves regulation of fluid flow in gastrovascular canals, propelled by cilia, and opening and closing of the central orifice [1, 23–25]. The numerous α-tubulin positive cilia are seen at the endodermal luminal epithelium of the canal (Fig. 6g).

### Distribution of peptidergic cells in *V. multiformis*

As reported in Hayakawa et al. (2022) [13], C-terminal amidation is a major modification in mature neuropeptides, and a number of amidated neuropeptides was identified from the pelagic ctenophore, *B. mikado*, through mass-spectrometry-based peptidomics. These peptides were found to be widely present in neurons and neurosecretory cells. Upon subjecting *B. mikado* larvae to these peptides, behavioral changes involving muscle contraction were induced, thereby substantiating their function as neuropeptides [13]. Notably, these peptide sequences showed a ctenophore-specific profile, while demonstrating broad conservation across ctenophore species, including *V. multiformis* [13] (Supplementary Table). We prepared antibodies against these neuropeptides and visualized morphology and distributions of peptide-expressing cells in *V. multiformis.* Synthetic peptide sequences used to produce antibodies are 100% identical between *B. mikado* and *V. multiformis*, except for RWFa (Supplementary Table).

#### VWYa

**Fig. 7:**
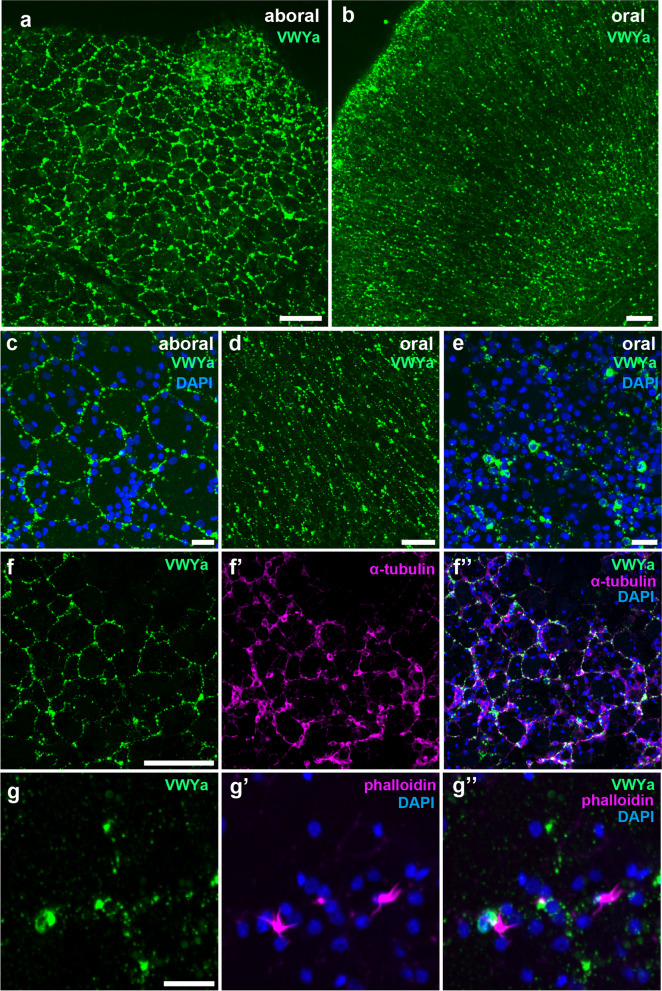
Distribution of VWYa-expressing neurons in subepithelial layers of *V. multiformis*. **a**–**e** Subepithelial neural networks on the aboral (**a**, **c**) and oral surfaces (**b**, **d**, **e**) are detected by immunofluorescent staining with anti-VWYa antibody. **f**-**f”** VWYa-positive neurons (green) develop polygonal neural networks in the aboral subepithelial layer that were partially overlapped by α-tubulin staining (magenta) in a reticular pattern. **g**–**g”** VWYa-positive neural net-like structures (green) and phalloidin-positive processes (magenta) on the aboral surface. Some of these processes (magenta) extend from VWYa-positive cell bodies (green). Scale bar, 50 μm (**a**, **b**, **d** and **f**) or 10 μm (**c**, **e**, and **g**)

**Fig. 8:**
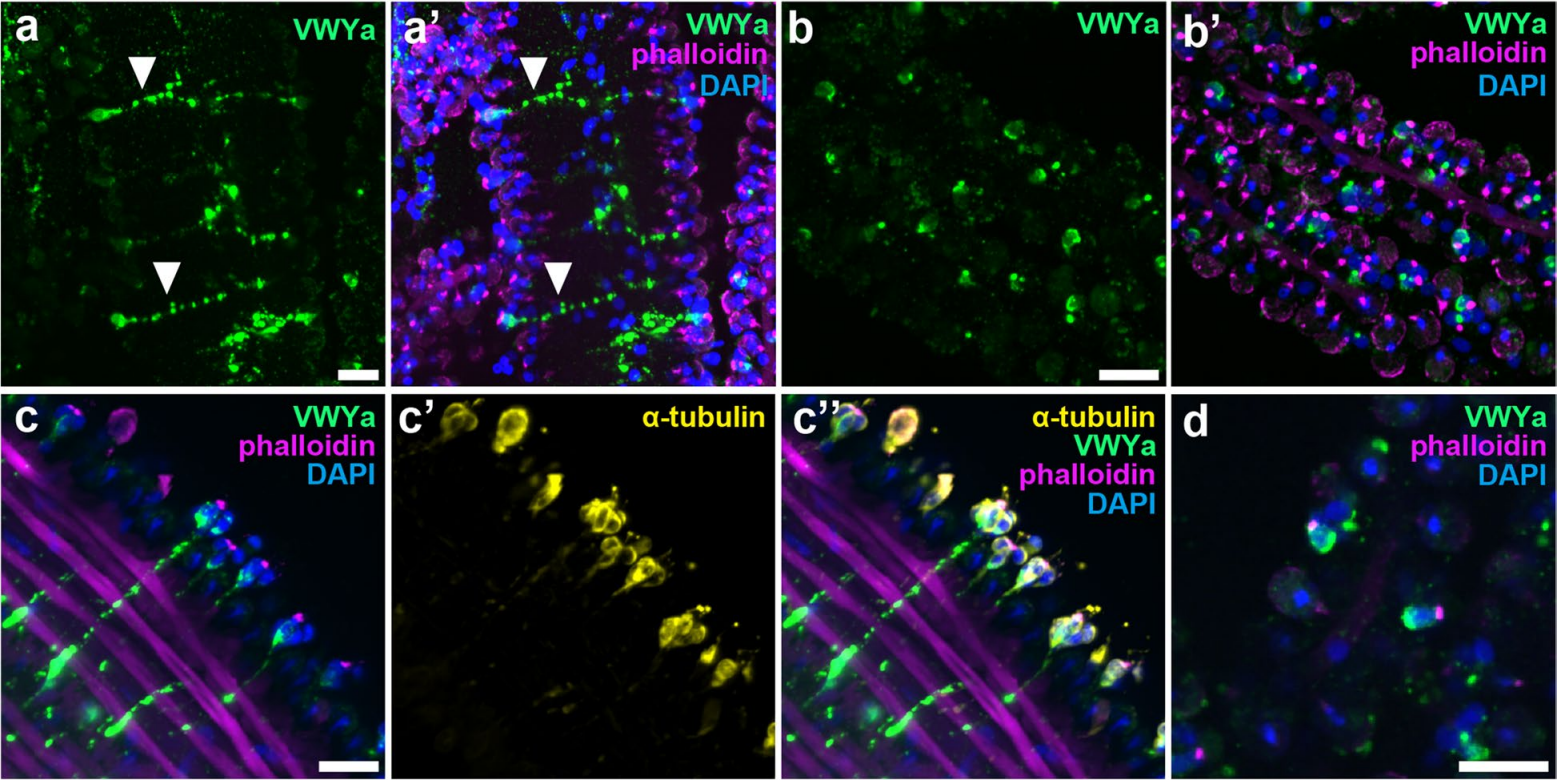
VWYa-positive neural architecture in a *V. multiformis* tentacle. **a**-**b’** The main trunk (**a**, **a’**) and tentilla (**b**, **b’**) were stained with anti-VWYa antibody (green), phalloidin (magenta), and DAPI (blue). Putative sensory cells on the surfaces were positive for VWYa and most of these cells have phalloidin-positive processes in their cell bodies. In the main trunk, neurite-like elongations of putative sensory neurons were positive for VWYa (arrowheads in **a** and **a’**). **c**-**c’’** Magnified images of VWYa-positive neurons in the main trunk. VWYa-positive neurons (green) which have phalloidin-positive processes (magenta) partially overlapped with α-tubulin-positive putative sensory cells (yellow). **d** VWYa-positive putative sensory cells (green) with phalloidin-positive processes (magenta) on the surface of the tentilla. Scale bar, 10 μm

A clear signal of VWYa staining was observed at the SNN of the aboral and oral surfaces of *V. multiformis* (Fig. 7a, b). On the aboral side, the meshwork exhibited a random polygonal pattern (Fig. 7a, c), whereas on the oral side, it extended radially and distally from the mouth opening (Fig. 7b, d, e). Double staining with anti-VWYa and anti-α-tubulin antibodies demonstrated that a portion of the α-tubulin-positive meshwork was also labeled by VWYa (Fig. 7f-f”). Cell bodies and neurites of VWYa-expressing neurons forming the SNN were frequently accompanied by bundles of phalloidin-positive processes nearby (Fig. 7g-g”). Although it is unclear whether these F-actin-rich structures are present in neurons or other nearby cells, it is possible that they are involved in signal perception and transmission. In *B. mikado*, VWYa-expressing neurons are distributed in the aboral organ and comb row in addition to the SNN [13], whereas it was undetectable in *V. multiformis*, which lacks comb rows. In the tentacles, putative sensory neurons/cells located on the surfaces of both the primary trunk and tentilla showed strong VWYa signals (Fig. 8). VWYa-positive cells on the trunk projected neurites toward the center of the trunk (Fig. 8a, c). Overlaying the signal with α-tubulin and VWYa indicated that only subsets of sensory neurons were VWYa-positive (Fig. 8c-c”).

#### NPWa

**Fig. 9:**
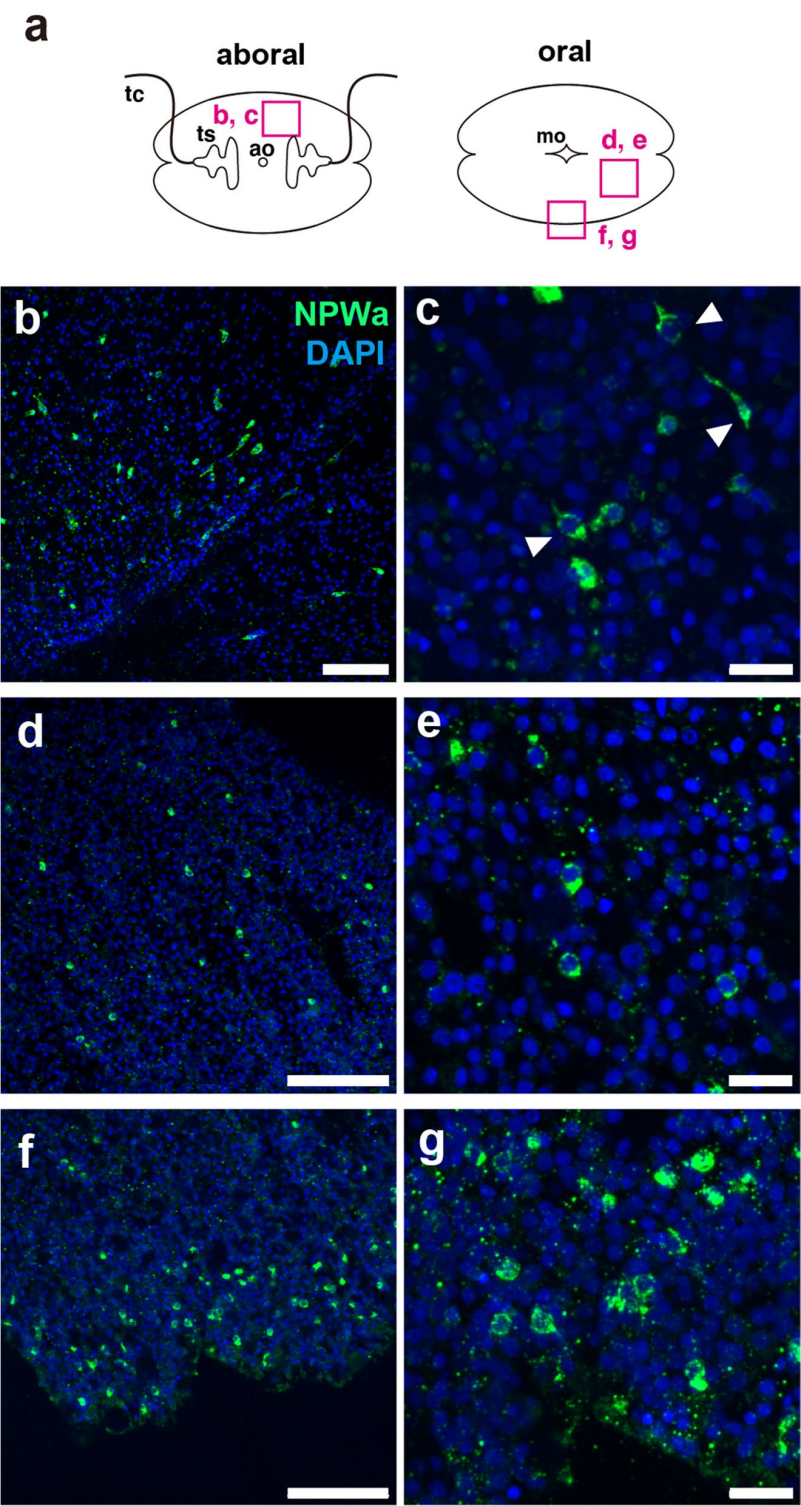
Distribution of NPWa-positive neurosecretory cells on *V. multiformis*. **a** A schematic representation of *V. multiformis.* Positions of the following images are indicated. **b**-**e** NPWa-positive cells were scattered on aboral (**b**, **c**) and oral (**d**, **e**) subepithelial surfaces. NPWa-positive cells on the aboral surfaces (**b**, **c**) displayed spindle-like or polygonal shapes and some of them had short processes (arrowheads in **c**), whereas cells on oral surfaces were spherical (**d, e**). **f**, **g** NPWa-positive spherical cells were enriched in the distal tissues of the body. Scale bar, 50 μm (**b**, **d** and **f**) or 10 μm (**c**, **e** and **g**)

We observed a sparse distribution of NPWa-positive cells in the subepithelial layer of both the aboral and oral sides of *V. multiformis* (Fig. 9). These cells were located in the same layer as VWYa-positive neural networks, although they exhibited neither neurites nor network-like structures. On the aboral surface, NPWa-positive cells displayed spindle-like or polygonal shapes with some cells possessing short processes (Fig. 9c), whereas at the oral side, spherical cells were observed (Fig. 9d–g). These two types of NPWa-positive cells were also found in *B. mikado* larvae, in which cells bearing processes are found in the subepithelial layer and spherical cells are observed in the pharynx [13]. Additionally, in *B. mikado*, NPWa-positive cells are also present beneath the comb rows and ciliary grooves [13]. However, as with VWYa-positive cells, no arrangement of NPWa-positive cells associated with comb rows and the aboral organ was observed in *V. multiformis*.

#### FGLa

**Fig. 10:**
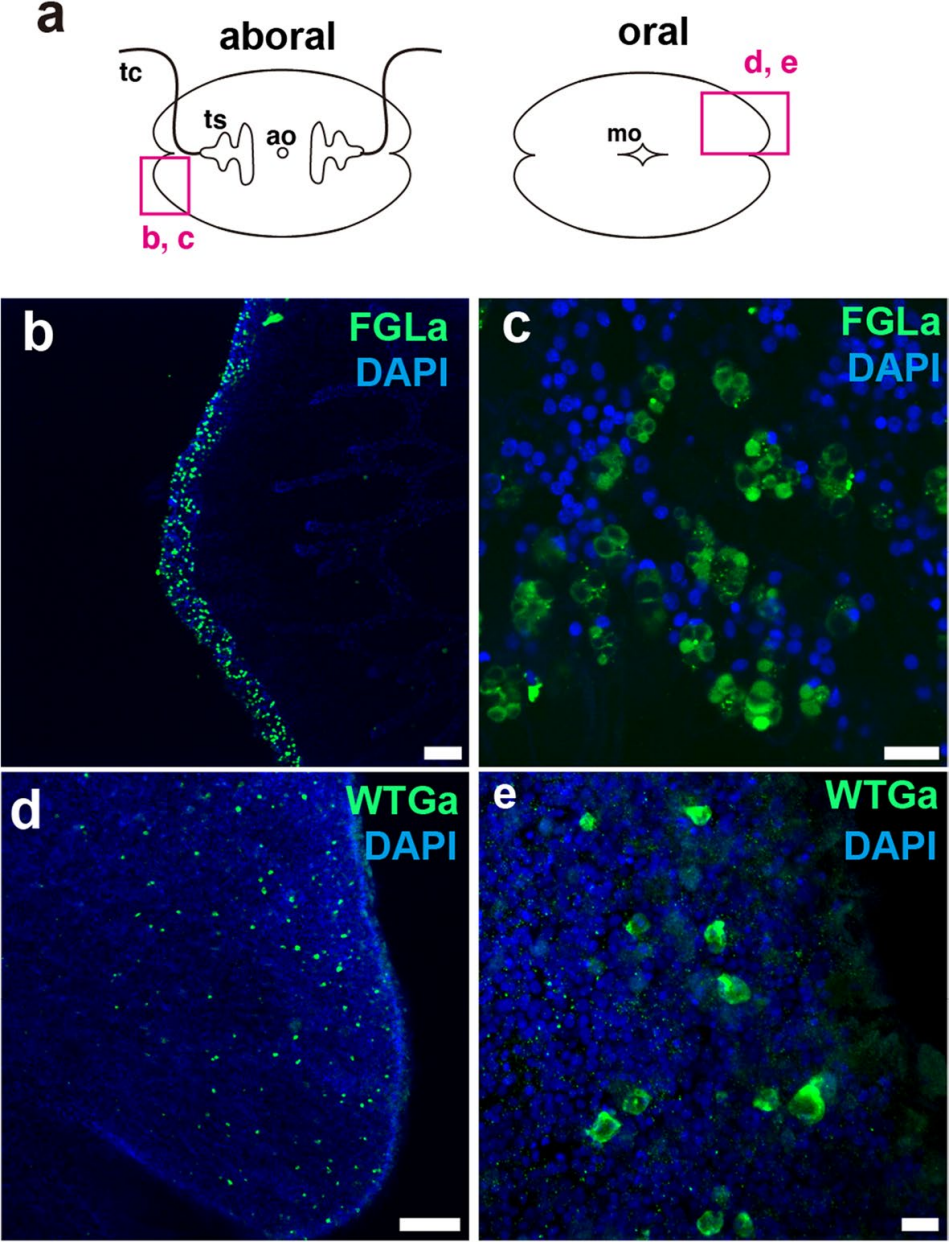
Distribution of FGLa- and WTGa-expressing cells in *V. multiformis*. **a** Positions of the following images in the animal are indicated in this schematic representation. **b**, **c** Distribution of FGLa-positive cells. They are enriched distal tissue of the body. **c** FGLa was detected as dots or signals surrounding large vacuolar structures in these cells. **d**, **e** Distribution of WTGa-positive cells. Small spherical cells were scattered on the oral surfaces. Scale bar, 100 μm (**b** and **d**) or 10 μm (**c** and **e**)

Strong FGLa-positive cells were localized in distal tissues of the *V. multiformis* body (Fig. 10b, c). In *B. mikado*, FGLa was prominently observed specifically in the region of the aboral organ [13], but no signals were observed around the aboral organ of *V. multiformis*.

#### WTGa

In *V. multiformis*, the signal of WTGa staining was not clear, but was detected in the sparsely distributed spherical cells of the oral surface (Fig. 10d, e). In *B. mikado*, WTGa was observed in the SNN [13], but these distribution patterns were absent in *V. multiformis*. Unlike *B. mikado*, *V. multiformis* did not exhibit WTGa distribution patterns in the floor plates of the aboral organ or nerve plexuses associated with comb rows [13].

## Discussion

### Differences in neuronal architecture between *V. multiformis* and pelagic species

In this study, we performed an immunohistochemical analysis aimed at revealing the neural and muscular organization of the benthic ctenophore, *V. multiformis*. Previous anatomical studies of pelagic ctenophores have identified distinct neural cell populations, including the subepithelial neural networks (SNN), mesogleal neurons, aboral neurons connecting ciliated grooves and comb rows, as well as tentacle sensory neurons [9, 21–26]. In adult *V. multiformis*, SNN and tentacle nerves resemble those of pelagic species. However, we observed clear differences involving loss or substantial alteration of other neuronal types. The aboral organ in *V. multiformis* is simple, with no neurite extension in either the polar field or the ciliated grooves. While the actual function of the aboral organ is still unclear, it is assumed to be involved in sensing gravity and body tilt, potentially regulating beating of comb cilia for swimming [30]. *V. multiformis* has comb rows in cydippid larvae [6, 28], suggesting possible existence of these neurons during the larval stage, followed by subsequent loss, concurrent with the disappearance of comb rows during metamorphosis. Neither tubulin staining nor neuropeptide staining revealed the presence of mesogleal neurons in adult *V. multiformis*. The function of mesogleal neurons in pelagic ctenophores is also unknown. It is conceivable that they may be present in *V. multiformis* larvae, if they perform functions related to swimming. These points require further investigation.

### Similarities and differences in muscular organization between *V. multiformis* and pelagic ctenophore species

**Fig. 11:**
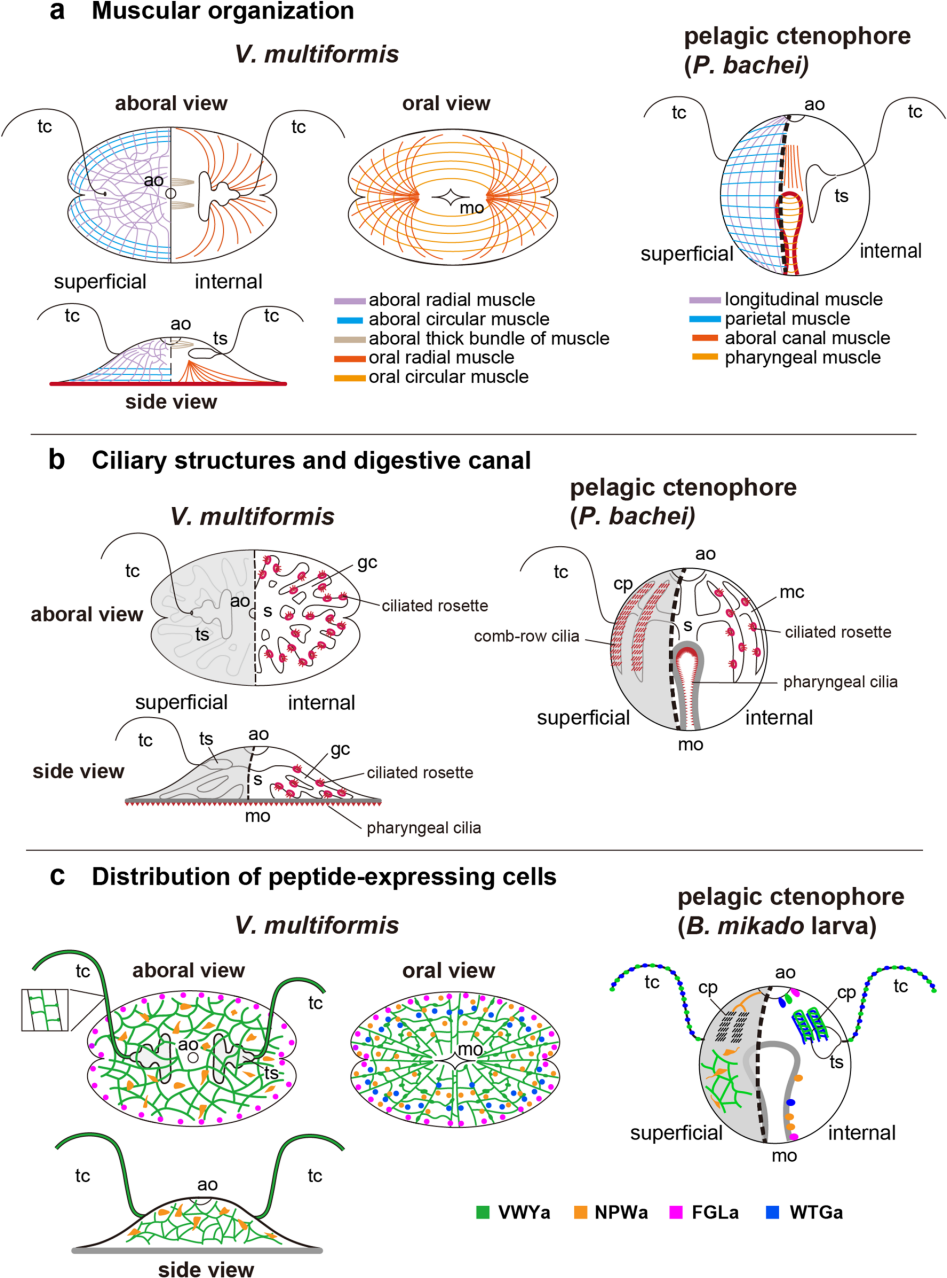
Summary of results in the schematic diagrams. Muscle organization (**a**), ciliary structures and digestive canal (**b**) and distribution of peptide-expressing cells (**c**) of *V. multiformis* and comparison with those of pelagic ctenophore. **a** Pattern of muscle organization in *V. multiformis* (left) and those of the pelagic ctenophore (*Pleurobrachia bachei*) (right). Muscles depicted with the same colors indicate their homology. **b** Organization of the digestive canals and the distribution of the characteristic ciliary structure, ciliated rosette, pharyngeal cilia, and comb-row cilia on *V. multiformis* (left) and the pelagic ctenophore (*P. bachei*) (right). The illustrations of *P. bachei* in **a** and **b** are reconstructed based on the description in Norekian and Moroz 2019a [23]. These cellular features are conserved across other pelagic ctenophore species [1, 2, 23–25, 31]. **c** Distribution of ctenophore peptide-expressing cells in bodies of *V. multiformis* (left) and a *B. mikado* larva (right). Distribution in *B. mikado* larvae are redrawn and modified from Hayakawa et al. (2022) [13]. Neurites are represented by lines and neurosecretory-like cells are represented by dots. ao, aboral organ; cp, comb plate; gc, gastrovascular canal; mc, meridional canal; mo, mouth; s, stomach; tc, tentacles; ts tentacle sheath

On the aboral surface of *V. multiformis*, we observed radially spreading muscles and circular muscles that exhibit a circular organization, intersecting perpendicularly with the radial muscles (Fig. 11a left, violet and blue lines). These muscular structures can be regarded as potential homologs to the longitudinal and parietal muscles found in pelagic species [23–25, 31] (Fig. 11a right, violet and blue lines). Additionally, circular muscles observed in the oral subepithelial layers may be homologous to the pharyngeal muscles of pelagic species [23–25] (Fig. 11a, pale orange lines). Among notable structural features of *V. multiformis* muscles, radial muscles stand out (Fig. 11a left, dark orange lines). These muscles extend from the central region of the body toward the oral side. Homologs of these muscles in pelagic species remain unclear. However, in certain species, such as *P. bachei*, *E. dunlapae* and *Mertensiidae* sp., thicker muscle fibers surrounding the aboral canal have been noted [23, 25], which may potentially correspond to the oral radial muscles of *V. multiformis* (Fig. 11a right, dark orange lines).

### Conservation of characteristic ciliary organization in *V. multiformis*

Ctenophores depend on functions of diverse ciliated structures [30]. The comb plate for swimming is representative of these (Fig. 11b). Although the non-swimming ctenophore, *V. multiformis*, does not have comb plates, it does possess other characteristic ciliated structures. We found that the oral epithelial surface is covered with such a ciliary structure, which consists of a bundle of cilia connecting the intracellular roots of F-actin bundles, and that this is similar to the ciliary structure on the inner surface of the pharynx of pelagic ctenophores [23–25, 34] (Figs. 5c and 11b). It was suggested that in *Beroe sp.*, these pharyngeal macrocilia are used as ciliary teeth to capture and tear prey [34]. Conservation of pharyngeal cilia in *V. multiformis* suggested that stiff cilia are used as spikes to attach to substrates in benthic, platyctenid species. In the gastrovascular canal of *V. multiformis*, we found long cilia covering the lumens and the ciliated rosette on the canal surface. Similar ciliated structures were observed in digestive canals of pelagic ctenophores [23–25] (Fig. 11b). The commonality of these ciliated structures in the gastrovascular canal suggests that regulating water flow by cilia of the inner surfaces and the ciliated rosette on the canal surface is responsible for transporting foods or debris in ctenophore digestive systems.

### Similar distribution of peptidergic systems between the oral surface of *V. multiformis* and the internal pharyngeal surface of pelagic ctenophores

From observations of adult morphology and metamorphosis of swimming larvae into the adult benthic form, it was concluded that oral surfaces of adult platyctenid species are homologous with the outer pharynx of other ctenophore species [4–6]. However, this was not examined at the cellular level. In the present study, we found similar ciliary structures on the oral epithelial surface of *V. multiformis* and on the pharyngal surface of pelagic ctenophores. Moreover, our study identified peptides FGLa, WTGa, and NPWa in spherical cells located on the oral surface of *V. multiformis*. This distribution closely mirrors their expression in spherical cells on the inner pharyngeal surface of *B. mikado* [13] (Fig. 11c). In the pharynx of *B. mikado*, FGLa-positive cells are located on the distal end, while NPWa- and WTGa-positive cells are relatively rostral [13]. These patterns are similar to the distribution of FGLa-positive cells in distal tissues and NPWa-positive cells in the inner region of the oral surface of *V. multiformis*. Taken together, these results support homology between the inner surface of the pharynx in pelagic species and the oral surface of platyctenid species, based on similarities of structure and functions of cells in these tissues.

### Commonality of peptide-expressing neurons among ctenophore species

We found the VWYa-positive SNN and NPWa-positive neurite-less cells on the subepithelial surface of *V. multiformis*. These distribution patterns closely resemble those found in *B. mikado* larvae [13], suggesting general conservation of these peptidergic systems throughout the Ctenophora. In tentacles of *V. multiformis*, VWYa signals were detected in sensory neurons on tentacle surfaces, and these cells extend neurites into the central fibers. VWYa-positive sensory neurons are also distributed in tentacles of *B. mikado* larvae, but no network pattern was observed [13]. This difference may be due to the immaturity of larval tentacles. The wide distribution of VWYa in networking neurons of SNN and tentacle nerves implies multiple and essential functions of this peptide in the ctenophore nervous system. For example, VWYa was detected in sensory neurons of both the body and tentacles, suggesting that VWYa peptides are used in systems that receive external stimuli.

On the other hand, the distribution of WTGa in the SNN detected in *B. mikado* was not observed in *V. multiformis*, suggesting a significant divergence in the function of this peptide between these species. In *B. mikado*, FGLa and WTGa were distributed in specific cells of the aboral organ [13], but this pattern was not observed in *V. multiformis*. NPWa was detected in ciliary grooves and comb rows [13], both of which are lost in *V. multiformis*. Peptides deployed in these neural structures of *B. mikado* may be involved in functions mediated by the aboral organ, such as gravity sensing and signal transmission for comb beating. Analyzing the distribution of cells expressing FGLa-, WTGa-, and NPWa neuropeptides in the larval aboral organ of benthic ctenophores may provide further insights into neural regulation of swimming behavior.

Recent anatomical studies of pelagic ctenophore species, along with findings of the present study on *V. multiformis*, indicate that all species examined so far possess SNNs [9, 21, 23–25]. Moreover, expression of VWYa in SNNs of both *B. mikado* and *V. multiformis* suggests a conserved VWYa-mediated function of the SNN among ctenophore species [13]. Recently, Burkhardt et al. (2023) [12] reported that neurons in the SNNs of *M. leidyi* larvae form a continuous plasma membrane, resulting in a syncytium without synaptic connections in this network. While it remains uncertain whether other ctenophore species have similar syncytial SNNs, this finding suggests that the pervasive neuropeptide transmission systems in metazoans are uniquely derived in ctenophores. Functionality and physiological attributes of syncytial SNNs are still unclear. However, the presence of the VWYa peptide in the syncytial SNN may enable rapid systemic secretion of VWYa neuropeptide under synchronous electrical excitation in the neural syncytium. A behavioral analysis, in which treatment of *B. mikado* larvae with VWYa peptide induced expansion of the entire body, suggests that the effector of VWYa includes the muscle or outer epithelium [13].

## Conclusion

In this study, we analyzed anatomical features of neurons and muscles of the benthic ctenophore, *V. multiformis*. Our findings demonstrated that *V. multiformis* has undergone a loss of certain neural structures, which are conserved in pelagic ctenophores, while the musculature is organized similarly to that of pelagic ctenophores. Furthermore, our detailed cytological analyses have revealed intriguing parallels between the oral surface of benthic ctenophores and the inner pharyngeal surface of pelagic ctenophores. Investigation of distribution patterns of neuropeptides in *V. multiformis* revealed a conserved distribution profile among benthic and pelagic ctenophore species. Notably, the peptide VWYa is distributed in subepithelial nerve nets and tentacle nerves, suggesting common, crucial functions in ctenophore neurons.

## Materials and methods

### Animals

*V. multiformis* were collected at Kiyoshi-Hiroshi sea-grape farm in Ginoza (Okinawa, Japan). They were cultured in 1.5 L of seawater with aeration and fed freshly hatched artemia three times per week.

### Immunohistochemical analysis

We used the monoclonal anti-α-tubulin antibody (T6074, SIGMA Aldrich) for tubulin staining. Polyclonal antibodies used for staining *V. multiformis* amidated peptides were originally prepared for *B. mikado* peptides. Samples of *V. multiformis* were placed on cover glasses in seawater. After animals attached to the glass, they were fixed with chilled 4% PFA in PBS at 4^o^C overnight. After washing with PBS with 0.1% Triton X100 (PBST) 3 × 15 min at room temperature and blocking with 1% bovine serum albumin (BSA) in PBS for 2 h, samples were incubated with primary antibodies diluted in 1% BSA in PBS at 4^o^C overnight. Dilution rates were as follows: anti-α-tubulin, 1:500; anti-VWYa, 1:100; anti-NPWa, 1:250; anti-FGLa, 1:300; anti-WTGa, 1:250. Then they were washed with PBST for 3 × 15 min at room temperature and incubated overnight at 4^o^C with 14 µM of 4’,6-diamidino-2-phenylindole (DAPI) and secondary antibodies and phalloidin in 1% BSA in PBS at the following dilutions: Alexa-488 conjugated goat anti-rabbit IgG (1:500) (111-545-003, Jackson ImmunoResearch); Alexa-488 conjugated goat anti-mouse IgG (1:500) (115-545-003, Jackson ImmunoResearch); Alexa-640 conjugated goat anti-rabbit IgG (1:500) (115-605-003, Jackson ImmunoResearch); Phalloidin-iFluor 555 conjugate (1:200) (20552, CAYMAN CHEMICAL). After washing with PBST 3 × 15 min, they were mounted with Slowfade Gold antifade reagent (S36937, Thermo Fisher Scientific). These fluorescent samples were observed, and images were recorded with a fluorescence microscopy system SD-OSR (Olympus) handled by Metamorph (Molecular Devices). Taken images were edited with ImageJ. Immunofluorescence images show a typical representative of 4–21 samples with similar results.

### Supplementary Information

**Additional file 1: Supplementary Table.** Amino acid sequences of amidated short peptides isolated from *B. mikado* and identical peptides from *V. multiformis*.

## Data Availability

Datasets used and/or analyzed during this study are available from the corresponding author on request.
