# Supplementary material for: Neuromuscular organization of the benthic ctenophore, Vallicula multiformis
Source: Zoological Lett. 2024 Jan 30;10:3. doi: 10.1186/s40851-024-00225-0 (PMC10826244; doi:10.1186/s40851-024-00225-0)
Supplement: Supplementary file 1 — Additional file 1: Supplementary Table. Amino acid sequences of amidated short peptides isolated from B. mikado and identical peptides from V. multiformis. [file 40851_2024_225_MOESM1_ESM.docx]

| peptide name | species | sequence |
| --- | --- | --- |
| NPWa | *B. mikado* | IGSDIKLVPG**A**GGNPWa |
|  | *V. multiformis* | IGSDIKLVPG**S**GGNPWa |
| VWYa | *B. mikado* | ARVYKGYNGGNRVWYa |
|  | *V. multiformis* | ARVYKGYNGGNRVWYa |
| WTGa | *B. mikado* | AKFSMSNYRGHKQGNRGWTGa |
|  | *V. multiformis* | AKFSMSNYRGHKQGNRGWTGa |
| FGLa | *B. mikado* | RA**Y**EESAGDENNQAFGLa |
|  | *V. multiformis* | RA**F**EESAGDENNQAFGLa |
| RWFa | *B. mikado* | **S**GTKFNKADYKSVGEGTR**R**WFa |
|  | *V. multiformis* | **A**GTKFNKADYKSVGEGTR**K**WFa |

Supplementary Table.

Amino acid sequences of amidated short peptides isolated from *B. mikado* and identical peptides from *V. multiformis*. Variable residues are shown in bold. Sequences of immunogen peptides used for antibody production are underlined. "a" in the sequence indicates the C-terminus amide group.
